# Profiling of Heterobranchia Sea Slugs from Portuguese Coastal Waters as Producers of Anti-Cancer and Anti-Inflammatory Agents

**DOI:** 10.3390/molecules23051027

**Published:** 2018-04-27

**Authors:** Nelson G. M. Gomes, Fátima Fernandes, Áurea Madureira-Carvalho, Patrícia Valentão, Alexandre Lobo-da-Cunha, Gonçalo Calado, Paula B. Andrade

**Affiliations:** 1 REQUIMTE/LAQV, Laboratory of Pharmacognosy, Departament of Chemistry, Faculty of Pharmacy, University of Porto, R. Jorge Viterbo Ferreira, no. 228, 4050-313 Porto, Portugal; mfgfernandes@gmail.com (F.F.); aurea.carvalho@iucs.cespu.pt (Á.M.-C.); valentao@ff.up.pt (P.V.); 2 IINFACTS—Institute of Research and Advanced Training in Health Sciences and Technologies, Department of Sciences, University Institute of Health Sciences (IUCS), CESPU, CRL, Central de Gandra Street, 1317, 4585-116 Gandra PRD, Portugal; 3 Departamento de Microscopia, Instituto de Ciências Biomédicas Abel Salazar (ICBAS), Universidade do Porto, 4050-313 Porto, Portugal; alcunha@icbas.up.pt; 4 Centro Interdisciplinar de Investigação Marinha e Ambiental (CIIMAR), 4450-208 Matosinhos, Portugal; 5 Departamento de Ciências da Vida, Universidade Lusófona de Humanidades e Tecnologia, 1749-024 Lisboa, Portugal; goncalo.calado@ulusofona.pt; 6 MARE NOVA. Faculdade de Ciências e Tecnologia, Universidade Nova de Lisboa, Departamento de Ciências e Engenharia do Ambiente, Campus da Caparica, 2829-516 Caparica, Portugal

**Keywords:** *Armina* spp., *Aglaja tricolorata*, sea slugs, fatty acids, PUFA, EPA, DHA, homarine

## Abstract

Bioprospection of marine invertebrates has been predominantly biased by the biological richness of tropical regions, thus neglecting macro-organisms from temperate ecosystems. Species that were not the object of studies on their biochemical composition include the Heterobranchia gastropods *Armina maculata*, *Armina tigrina* and *Aglaja tricolorata*, inhabitants of the Portuguese Atlantic coastal waters. Here, we present for the first time the fatty acid profile of neutral lipids and homarine content of these three species. Qualitative and quantitative differences in the fatty acid content among species points to the existence of a fatty acid profile of neutral lipids, particularly of each genus. The results from cytotoxicity assays, using the acetonic extracts of the gastropods on human gastric adenocarcinoma (AGS) and human lung adenocarcinoma (A549) cell lines, revealed a pronounced cytotoxic effect of the *A. tigrina* extract on both cell lines (IC_50_ values of 68.75 and 69.77 μg mL^−1^ for AGS and A549, respectively). It is worth noting the significant reduction of NO levels in LPS-challenged RAW 264.7 macrophages exposed to *A. tricolorata* extract, at concentrations as low as 125 μg mL^−1^.

## 1. Introduction

The increasing number of reports on the structural variety and pharmacological properties of naturally occurring compounds obtained from marine invertebrates [1,2] has been driving the focus on several taxonomic groups, namely Heterobranchia mollusks [3,4].

Driven by the richer biological diversity compared to temperate regions, marine organisms from tropical ecosystems have been preferentially targeted as sources of therapeutically useful chemical agents [5]. Despite the geographical trends, bioprospection of temperate environments has been rewarding, leading to the discovery of a significant number of compounds from marine invertebrates [6]. Several species of nudibranchs, such as *Armina* spp., are widely distributed in the Mediterranean and Atlantic coasts, namely *Armina maculata* Rafinesque, 1814 and *Armina tigrina* Rafinesque, 1814 [7]. The cephalaspidean *Aglaja tricolorata* Renier, 1807 shares a similar geographical distribution, being also recorded along the Atlantic coast [8]. Despite their ubiquitous distribution, inhabiting sub-tropical and temperate shores [9], the above-mentioned species are considered scarce and/or seldom found, the chemical investigation of both genera being virtually absent [10]. There is a single study on the isolation of briarane and cembrane diterpenoids from *A. maculata*, and their subsequent identification in its prey *Veretillum cynomorium* [11].

As building blocks of several biologically relevant molecules, fatty acids act as biochemical mediators, energy sources and structural components [12], being obtained through heterotrophic and autotrophic pathways. They are frequently used as chemotaxonomic and chemo-ecological markers in some taxonomic groups [13]. While the fatty acid profile of a marine invertebrate is predominantly determined by its dietary sources, environmental and biotic factors can influence on the profile, providing valuable information on its ecological traits and supporting their chemotaxonomic discrimination [13,14]. Several experimental and epidemiological studies have demonstrated that specific classes of fatty acids, namely polyunsaturated fatty acids (PUFA), exhibit anti-cancer effects and immunoactive functions [15,16], playing also a pivotal role on the inflammatory process as precursors of eicosanoids [17]. Given the regression of the shell, and lack of an acquired immune system, some mollusks have evolved alternative mechanisms of defense, being active producers of bioactive metabolites for circulation in the hemolymph, as well as toxic and deterrent secretions, frequently containing molecules exhibiting anti-inflammatory and cytotoxic properties [18]. In addition to fatty acids, the biosynthetic machinery of marine macro-organisms enables the production of secondary metabolites falling into a wide range of structural classes.

With the current study, we aimed to investigate the chemical profiles of *A. maculata*, *A. tigrina* and *A. tricolorata*, providing further insights on their metabolome. Considering that Heterobranchia sea slugs rely mainly on chemical defenses through the production of intrinsically active molecules, it was also our aim to assess their cytotoxicity towards human gastric (AGS) and human lung (A549) adenocarcinoma cell lines, as well as the anti-inflammatory potential, evaluated through the interference with the production of nitric oxide (NO) in lipopolysaccharide (LPS)-challenged RAW 264.7 macrophages.

## 2. Results

### 2.1. Chemical Characterization

#### 2.1.1. Fatty Acid Profiles

**Figure 1:**
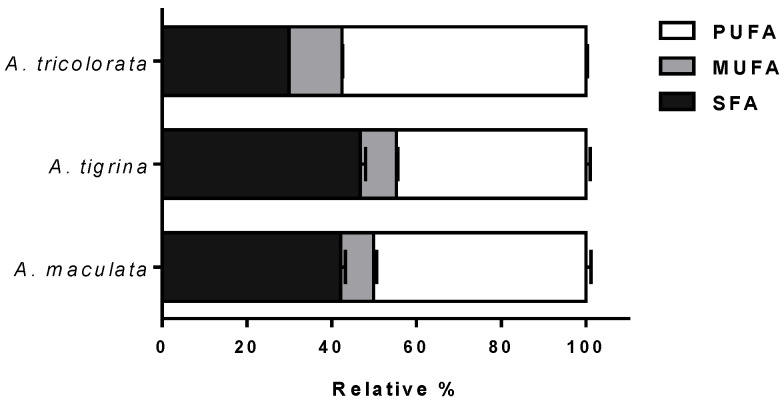
Relative content of SFA, MUFA and PUFA in the analyzed extracts. The results correspond to the mean ± SD of three independent determinations.

As shown in Table 1, specimens under study are characterized by a complex fatty acid profile, their quantification revealing total fatty acid concentrations ranging from 136.21 to 165.81 μg mg^−1^ dry extract. In *A. maculata* and *A. tigrina* extracts, 23 and 26 fatty acids were characterized, respectively, while, in *A. tricolorata* extract, 18 fatty acids were identified, indicating clear differences between the two genera. Concerning the main constituents, the fatty acids contents of *A. maculata* and *A. tigrina* were rather similar, C16:0, C18:0, C20:4*n*-6, and C20:5*n*-3 being identified as the main components (>10%, each), accounting for approximately 63% of the total fatty acid content. Nonetheless, the saturated fatty acids (SFA) C21:0 and C22:0, and the PUFA C18:2*n*-6t were detected only in *A. tigrina* extract. While C16:0, C18:0 and C20:5*n*-3 were also identified as main components, the acetone extract of *A. tricolorata* exhibited high contents of C20:2*n*-6 (ca. 12%) and C22:6*n*-3 (ca. 13%). Moreover, distinct proportions of the different classes of fatty acids was also evidenced for the two genera (Figure 1).

*A. maculata* and *A. tigrina* presented a similar qualitative profile concerning the SFA, monounsaturated fatty acids (MUFA) and PUFA content, while *A. tricolorata* was significantly richer in PUFA (ca*.* 57%). The cephalaspidean *A. tricolorata* exhibited a significantly higher proportion of *n*-3 PUFA (*n*-6/*n*-3 < 1), in contrast with the nudibranchs *A. maculata* and *A. tigrina* with a *n*-6/*n*-3 ratio higher than 1.75 (Table 1).

**Figure 2:**
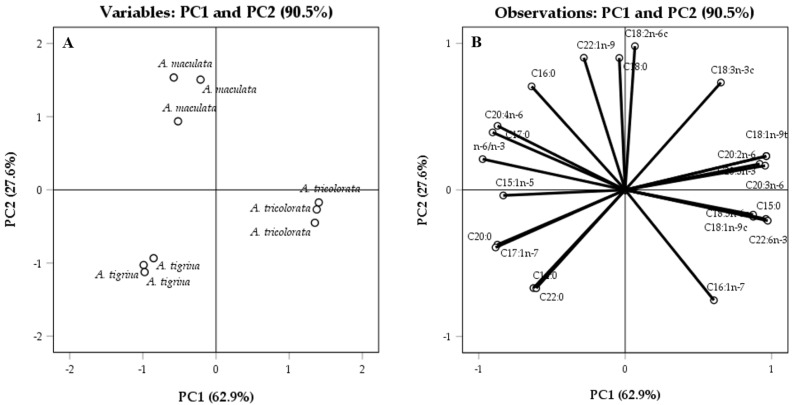
Projection of *A. maculata*, *A. tigrina*, and *A. tricolorata* (**A**); and loadings by fatty acid composition (**B**) into the plan composed by the principal components PC1 and PC2 containing 90.5% of the total variance for sample fatty acid composition. Eigen values obtained for PC1 and PC2 were 13.8 and 6.1, respectively.

To identify possible dissimilarities between the Heterobranchia sea slugs, PCA was applied. PCA was performed for all the species using the content (μg mg^−1^ dry extract) of each fatty acid and *n*-6/*n*-3 ratio of each sample as variables. As evidenced in Figure 2A, this model allowed separating the three species according to their fatty acid content; the two scores obtained from the latent vectors accounted for all registered variations (90.5%), PC1 accounting for 62.9% of the variance and PC2 for 27.6% (Figure 2).

#### 2.1.2. Homarine

HPLC-DAD analysis allowed finding homarine in the three acetone extracts, being present in higher amounts in both *A. maculata* and *A. tricolorata* (Table 2). Quantitative analysis demonstrated significant differences between the acetone extracts obtained from *A. maculata* and *A. tricolorata* (*p* < 0.001), homarine being found at 969.74 ± 43.90 and 1237.15 ± 35.08 μg g^−1^ dry extract, respectively.

### 2.2. Impact on AGS and A549 Cancer Cells

Prompted by the fatty acids composition and by the chemo-defensive metabolite homarine, we aimed to investigate the potential cytotoxicity of the extracts towards the human cancer cell lines AGS and A549.

**Figure 3:**
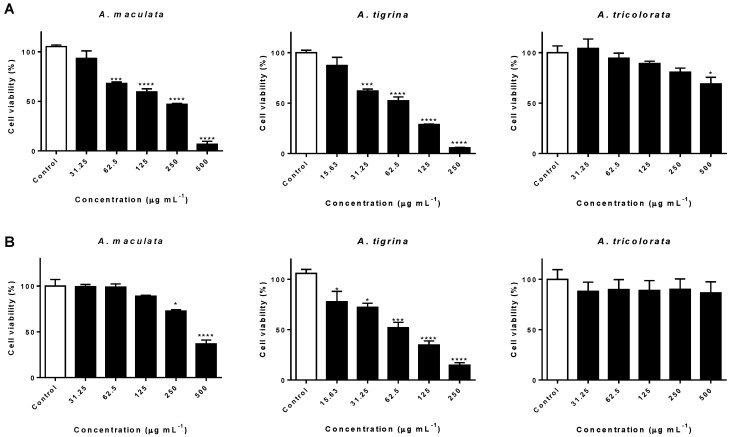
Effects of *A. maculata*, *A. tigrina* and *A. tricolorata* on the viability of human gastric adenocarcinoma (AGS) (**A**) and human lung adenocarcinoma (A549) (**B**) cells upon 24 h treatment. The results correspond to the mean ± SEM of three independent experiments, performed in triplicate. Statistical significance: * *p* < 0.05, *** *p* < 0.001, and **** *p* < 0.0001.

While *A. tricolorata* extract caused a significant cytotoxic effect (*p* < 0.05) only at the highest concentration tested (500 µg mL^−1^), treatment with extracts of the Arminidae *A. maculata* and *A. tigrina* clearly interfered on the viability of AGS cells in a concentration-dependent manner, displaying IC_50_ values of 220.66 and 68.75 µg mL^−1^, respectively (Figure 3A). Notably, the acetone extract of *A. tigrina* markedly reduced the cell viability rate (*p* < 0.0001) down to 5.89 ± 0.70% at 250 µg mL^−1^ (Figure 3A).

Evaluation of the potential interference on the viability of A549 cells revealed a cytotoxic effect upon treatment with *A. tigrina* extract (IC_50_ = 69.77 µg mL^−1^), leading to a reduction of more than 50% on cell viability in concentrations ranging from 125 to 250 µg mL^−1^ (Figure 3B). Exposure to *A. maculata* extract was also accompanied by significant cytotoxicity at 500 µg mL^−1^ (*p* < 0.0001), while no noticeable interference on cell viability was caused by *A. tricolorata* (Figure 3B).

### 2.3. Impact on RAW 264.7 Macrophages and Interference on NO Levels

Depending on both local and spatial concentrations, NO production can cause a duality of effects. Production of NO is associated with beneficial effects, such as antibacterial and anti-tumor, while its overproduction is associated with inflammatory states [19]. Since fatty acids, particularly *n*-3 PUFA, have been widely reported as anti-inflammatory agents, influencing membrane function and several cellular processes [17], another aim of the current study was to assess the potential anti-inflammatory effects of the three extracts in LPS-challenged RAW 264.7 macrophages.

**Figure 4:**
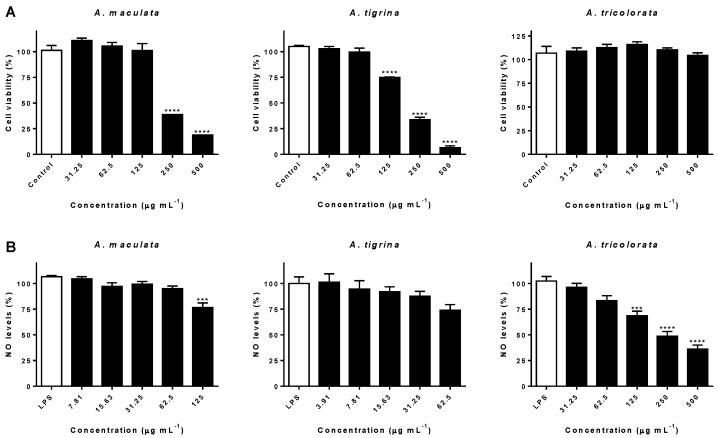
Effects of *A. maculata*, *A. tigrina* and *A. tricolorata* on RAW264.7 cell viability (**A**), and effects on NO levels in LPS-stimulated RAW 264.7, cells upon 24 h treatment (**B**). The results correspond to the mean ± SEM of three independent experiments, performed in triplicate. Statistical significance: *** *p* < 0.001 and **** *p* < 0.0001.

To discard the possibility of the inhibitory effects being correlated with a cytotoxic effect upon RAW 264.7 cells, influence on cell viability was firstly assessed. No noticeable interference was observed after treatment with *A. tricolorata* at concentrations as high as 500 µg mL^−1^ (Figure 4A). In contrast, both *A. maculata* and *A. tigrina* caused a significant decrease on the viability of RAW 264.7 macrophages, at 250 (*p* < 0.0001) and 125 µg mL^−1^ (*p* < 0.0001), respectively.

Therefore, the higher working concentrations selected for the determination of NO levels after exposure to *A. maculata*, *A. tigrina* and *A. tricolorata* were 125, 62.5 and 500 µg mL^−1^, respectively.

Exposure to *A. maculata* acetone extract led to a significant reduction in NO production (ca. 25%), at the highest concentration tested (Figure 4B). Similarly, at concentrations ranging from 3.91 to 62.5 μg mL^−1^, *A. tigrina* did not cause a noticeable interference in NO production (Figure 4B). Concerning *A. tricolorata*, a reduction of NO levels down to 68.52 ± 8.08% was observed at a concentration of 125 µg mL^−1^, higher concentrations markedly interfering with those levels (*p* < 0.0001) (Figure 4B).

### 2.4. Screening for Antimicrobial Activity

To further characterize the potential biological properties of the extracts under study, we have screened the samples for antimicrobial activity against representative species of bacteria and one yeast grown. The bacteria tested were *Escherichia coli*, *Staphylococcus aureus*, *Pseudomonas aeruginosa*, while the yeast was *Candida albicans*. Up to the concentration 2.5 mg mL^−1^, no activity was found, with solubility issues hindering the assessment of higher concentrations.

## 3. Discussion

### 3.1. Chemical Characterization

#### 3.1.1. Fatty Acid Profiles

The results obtained with the current study constitute the first record on the fatty acid content of both genera, with potential ecological significance in distinguishing Heterobranchia sea slugs [14]. Identification of C16:0, C18:0 and C20:4*n*-6 as the main constituents in the species under study was expected, being in clear agreement with previous reports on the fatty acid content of nudibranchs [20]. While marine invertebrates possess genes encoding enzymes involved in the endogenous production of PUFA, the majority originates from trophic upgrading, being almost exclusively produced by microalgae, bacteria and heterotrophic protists [21]. Consequently, the identification of the photosynthetic symbiont markers C18:3*n*-6c, C20:5*n*-3 and C22:6*n*-3 [22] was not surprising, C20:5*n*-3 and C22:6*n*-3 being found in higher amounts in *A. tricolorata* (ca. 15% and 13% of total fatty acids, respectively). Relevantly, it was also observed a significant content in odd-chain fatty acids, usually originating from symbiotic bacteria [22]. C17:0 was identified on the three species of sea slugs, accounting for nearly 10% of the total fatty acid content in both *Armina* specimens, and ca. 6% in *A. tricolorata*, while C15:0 was detected in minute quantities (Table 1).

Being evident that dietary sources impact fatty acids content of marine invertebrates, it is relevant to refer that the three species are carnivorous but differ on their feeding behavior. Despite the reports on the dietary habits of *Aglaja felis*, feeding upon foraminiferans, the specific diet of *A. tricolorata* is unknown [8]. Concerning the Arminidae under study, *A. maculata* often feeds on the pennatulacean *Veretillum cynomorium* [23]. Curiously, temporal analysis on the variation of fatty acids content in *V. cynomorium* specimens collected at the same site (Caldeira de Tróia), revealed that it is predominantly constituted by C16:0, C18:0, C20:4*n*-6 and C20:5*n*-3 (ca. 37–44% of total fatty acids) [24], thus evidencing similarity with the profile of *A. maculata* that presented the same major compounds (Table 1). On the other hand, *V. cynomorium* exhibits a content in *n*-3 at least one order of magnitude higher than *n*-6 [24], contrary to *A. maculata* (Table 1). Marine organisms are generally characterized by the predominance of *n*-3 PUFA [25], being particularly rich in the common marine PUFA C20:5*n*-3 and C22:6*n*-3, usually constituting nearly half of the total fatty acid content in molluscs [20]. While unusual in marine invertebrates, the higher levels of *n*-6 relative to *n*-3 in both *Armina* (Table 1) were in agreement with previous studies on the fatty acid profile of nudibranch sea slugs, where *n*-6 PUFA were dominant [26]. 

PCA confirmed the chemo-ecological significance of the fatty acid profiles, allowing identifying intergenera and interspecies differences, based on the content on specific fatty acids (Figure 2). The highest content of C16:1*n*-7, C18:1*n*-9c, C18:1*n*-9t, C20:2*n*-6, C18:3*n*-6c, C20:3*n*-6 and C20:5*n*-3 (Table 1) dictated the positioning of *A. tricolorata* on the positive side of the plan, thus evidencing their role as distinguishing fatty acids between the genera *Armina* and *Aglaja* (Figure 2A). It is also worth noting *n*-6/*n*-3 as a distinguishing parameter, objectively contributing to the separation between the two genera, as evidenced in Figure 2A,B. Relevantly, the absence of C15:1*n*-5, C17:1*n*-7 and C22:0 in *A. tricolorata*, and their presence in significant amounts on the extract obtained from *A. tigrina*, allowed separating these two species along PC1 (Figure 2A,B). 

Despite the similar quantitative profiles, the reason they appear in the negative part of PC1 is that both Arminidae were distinguished from each other along PC2: given the levels of C22:0 and the *n*-7 acids C16:1*n*-7 and C17:1*n*-7, *A. tigrina* is positioned in the negative axis, *A. maculata* appearing in the positive plan of PC2 based on its levels of C16:0, C17:0, C18:0, C22:1*n*-9 and the *n*-6 acids C18:2*n*-6c and C20:4*n*-6 (Figure 2A,B and Table 1).

#### 3.1.2. Homarine

According to König and colleagues, homarine is ubiquitously found in Cladobranchia, namely in *Armina* spp. [27], its detection being expected in *A. maculata* and *A. tigrina*. However, while uncommonly found in other members of the Heterobranchia [28], in agreement with our results, the same authors reported the detection of homarine in *A. tricolorata* [27]. Several roles have been described for the cyclic betaine derivative, considered to be a chemical defense tool in marine invertebrates, acting as both predator deterrent and antifouling agent [28].

### 3.2. Impact on AGS and A549 Cancer Cells

A large body of evidence indicates that certain *n*-3 fatty acids can selectively interfere with the viability of tumor cells, exerting their effects through several mechanisms, such as generation of reactive oxygen species (ROS), lipid peroxidation, activation of caspases and peroxisome proliferator-activated receptors (PPAR), and modulation of oncogenes expression [29,30].

Identified in relevant amounts in the extracts obtained from the three species (Table 1), mainly in *A. tricolorata*, docosahexaenoic (C22:6*n*-3) and eicosapentaenoic (C20:5*n*-3) have been reported as pro-apoptotic agents in a wide range of tumors, namely in gastric and lung cancers [31]. As recently reported by Gao et al. [32], C22:6*n*-3 displays a potent in vitro anti-proliferative effect towards AGS cells in concentrations ranging from 7.5 to 45 µg mL^−1^. Since this compound is found in the same range of concentrations on the extract of *A. tricolorata* (10.64 ± 0.77 µg mL^−1^) at the highest concentration tested, it is plausible to hypothesize that C22:6*n*-3 plays a role on the observed cytotoxic effects (Figure 3A). Concerning the possible role of homarine in the cytotoxic effects observed towards AGS cells upon treatment with *A. maculata* and *A. tricolorata*, we have previously demonstrated that this alkaloid could only interfere in a superior range of concentrations [33], thus neglecting its contribution.

Previous reports on the in vitro cytotoxic effects of C20:5*n*-3 and C22:6*n*-3 towards A549 cancer cells may partially explain the observed effects upon treatment with the extracts under study (Figure 3B). Yao et al. [34] indicated that C20:5*n*-3 and C22:6*n*-3 induced apoptosis and autophagy, suggesting that both mechanisms mediate the cytotoxicity towards A549 cells. Zajdel and co-workers reported a concentration-dependent effect attributed to the induction of oxidative damage [35]. The ability of C20:4*n*-6, identified in high amounts in both *A. maculata* and *A. tigrina* extracts, to interfere with A549 cell growth, in both concentration- and time-dependent manners, was also reported [36].

Cancer cells are generally characterized by a deficit in PUFA, mainly in C20:4*n*-6, C20:5*n*-3 and C22:6*n*-3 [37]. The observed effects towards the human cancer cell lines AGS and A549 may partially derive from the high contents of both C20:4*n*-6 and C20:5*n*-3, mainly in *Arminia* spp., since the exposure of tumor cells to certain PUFA leads to apoptotic cell death [37]. On the other hand, considering the qualitative and quantitative similarity between the fatty acid profiles of *A. maculata* and *A. tigrina* (Table 1), as well as the significantly stronger cytotoxic effects observed upon treatment with the latter, it seems plausible to consider that other classes of metabolites may contribute to the effects observed on the viability of AGS and A549 human cancer cell lines.

### 3.3. Impact on RAW 264.7 Macrophages and Interference on NO Levels

SFA and *n*-6 PUFA have been identified as inflammatory inducers, while *n*-3 PUFA, particularly C20:5*n*-3 and C22:6*n*-3, have been widely reported for their anti-inflammatory properties [38], namely on the efficient reduction on the production of NO in RAW 264.7 macrophages [39]. Additionally, also the *n*-3 PUFA C18:3*n*-3c exhibited a strong ability to interfere with NO levels in the same experimental model [40], being identified in considerable amounts on the acetone extracts of the sea slugs under study (Table 1). Due to the presence of C20:5*n*-3 and C22:6*n*-3 acids in high amounts on *A. tricolorata* extract, and in a similar range of concentrations as those specified in Ambrozova et al. [41], we can hypothesize that both *n*-3 PUFA play a role on the decrease of NO levels (Figure 4B).

The similar fatty acid profiles of both Arminidae can justify the comparable interference on the production of NO, namely concerning the amounts of the above mentioned fatty acids. However, the significant and distinctive effects on NO levels upon treatment with *A. tricolorata* may be explained by the higher content of C22:6*n*-3, identified as a major component, as well as by the low *n*-6/*n*-3 ratio (Table 1).

We have previously demonstrated that homarine failed to significantly interfere with the overproduction of NO in LPS-stimulated RAW 264.7 macrophages [33]. While the effects on NO production upon treatment with *A. tricolorata* may be partially due to its fatty acid profile, the contribution of other compounds cannot be excluded.

## 4. Materials and Methods

### 4.1. General Chemicals and Materials

Acetone EMSURE^®^, acetonitrile LiChrosolv^®^, methanol LiChrosolv^®^ and tetrahydrofuran LiChrosolv^®^ were purchased from Merck (Darmstadt, Germany). Purified water was treated in a Milli-Q water purification system (Millipore, Bedford, MA, USA). Anhydrous sodium sulfate, boron trifluoride (BF_3_)–methanol solution, dimethyl sulfoxide (DMSO), isooctane, phosphoric acid (H_3_PO_4_), potassium hydroxide (KOH), *N*-[naphth-1-yl]ethylenediamine dihydrochloride, sulfanilamide, and thiazolyl blue tetrazolium bromide (MTT) were from Sigma-Aldrich (St. Louis, MO, USA). Whatman^®^ Grade 1 filtration paper was obtained from Sigma-Aldrich (St. Louis, MO, USA) and 0.45 µM pore size membrane from Millipore (Bedford, MA, USA).

Murine macrophage-like cell line RAW 264.7 was from the American Type Culture Collection (LGC Standards S.L.U., Barcelona, Spain), AGS cells were from Sigma-Aldrich (St. Louis, MO, USA), and A549 cells were from European Collection of Authenticated Cell Cultures (ECACC). Dulbecco’s Modified Eagle Medium (DMEM), DMEM/F12, heat-inactivated fetal bovine serum (FBS) and Pen Strep solution (penicillin 5000 U mL^−1^ and streptomycin 5000 µg mL^−1^) were from GIBCO, Invitrogen (Grand Island, NY, USA). LPS was from *Escherichia coli* (Sigma-Aldrich, St. Louis, MO, USA).

Spectrophotometric determinations were performed in a Multiskan™ GO microplate spectrophotometer (Thermo Fisher Scientific Oy, Vantaa, Finland).

### 4.2. Animal Material and Extraction

Specimens of *A. maculata*, *A. tigrina* and *A. tricolorata* were hand collected during low tide between May and June 2016 in Praia de Tróia-Caldeira (Setúbal, Portugal). Samples were immediately placed in a container with sea water and transported to the laboratory, underwent a 48-h fasting period, being subsequently washed with a saline solution and kept at −20 °C until use. Voucher specimens of *A. maculata* (MI17), *A. tigrina* (MI18) and *A. tricolorata* (MI20) were deposited at the Laboratory of Pharmacognosy, Faculty of Pharmacy, University of Porto. Extracts were obtained in agreement with a slightly modified version of the protocol by Ebada et al. [42]. Briefly, animal specimens were sliced, extracts being obtained from the resulting biomass of each species (63.90, 8.21 and 20.60 g, for *A. maculata*, *A. tigrina* and *A. tricolorata*, respectively) through maceration with acetone, under magnetic stirring (500 rpm) at 30 °C. The resulting extracts were filtered through Whatman^®^ Grade 1 filtration paper and concentrated to dryness under reduced pressure (Büchi Labortechnik AG Rotavapor^®^ R-215, Flawil, Switzerland), yielding 836.8, 101.0 and 404.8 mg of acetonic extracts for *A. maculata*, *A. tigrina* and *A. tricolorata*, respectively. Consequently, the yields of extraction with acetone from *A. maculata*, *A. tigrina* and *A. tricolorata* were 1.31%, 1.23% and 1.97%, respectively.

### 4.3. Chemical Characterization

#### 4.3.1. Standards

A standard solution of fatty acid methyl esters (Supelco^®^ 37 Component FAME mix; CRM47885), was purchased from Sigma-Aldrich (Bellefonte, PA, USA). Homarine was synthesized as recently described in Silva et al. [33].

#### 4.3.2. GC-FID Qualitative and Quantitative Analysis

Thirty milligrams of each dry extract were hydrolyzed with 1 mL of KOH methanol solution (11 g L^−1^), at 90 °C, for 10 min. Extracts were derivatized according to the classical method by Metcalfe and Schmitz [43]. The free fatty acids originally present and those resulting from alkaline hydrolysis were derivatized to their methyl esters (FAMEs) with 1 mL of BF_3_-methanol solution (10%), at 90 °C, for 10 min. FAMEs were purified with 2 × 6 mL of isooctane and anhydrous sodium sulfate was added to assure the total absence of water. The resulting extracts were evaporated to dryness under a stream of nitrogen and dissolved in 200 µL of isooctane.

Purified FAME extracts were analyzed in a Finnigan Focus GC apparatus (Thermo Fisher Scientific, Waltham, MA, USA), equipped with a flame ionization detector (FID) and a VF-5 ms (30 m × 0.25 mm × 0.25 µm) column (Varian BV, Middelburg, The Netherlands). Injector and detector were maintained at 250 °C, and the oven heating program consisted on a linear increase of column temperature from 40 to 300 °C, at a rate of 5 °C min^−1^. Derivatized extracts (1 μL) were injected in triplicate. Quantification of each FAME was achieved from the equations of linear regression of the respective standard prepared in isooctane. The linearity range of the method was assessed by building calibration curves using five different concentration levels of the analytes, in triplicate, according to the range of concentrations found in the samples. The limit of detection (LOD) and limit of quantification (LOQ) were determined from calibration curve data, according to the following equations: LOD = 3.3σ/S and LOQ = 10σ/S, where *σ* is the residual standard deviation of the linear regression, and *S* is the slope of the regression line.

#### 4.3.3. HPLC-DAD Qualitative and Quantitative Analysis

The dried residues of *A. maculata* (75 mg mL^−1^), *A. tigrina* (50 mg mL^−1^) and *A. tricolorata* (50 mg mL^−1^) extracts were redissolved in tetrahydrofuran, filtered through 0.45 µM pore size membrane, and analyzed in triplicate on an analytical HPLC unit (Gilson Medical Electronics, Villiers le Bel, France), using a 250 × 4.6 mm, 5 µm, Spherisorb ODS2 80 Å RP-C18 column (Analytical Cartridge, Part No. PSS839540, Waters, Dublin, Ireland). Gradient conditions and mobile phase were the same as those described in Silva et al. [33]. The injection volume was 20 µL and the elution was performed at 25 °C. Detection was achieved with an Agilent 1100 series diode array detector (DAD) (Agilent Technologies, Waldbronn, Germany). Spectral data from all peaks were accumulated in the range of 200–700 nm and chromatograms were recorded at 280 nm. Data were processed on a Clarity software system, version 5.04.158 (DataApex Ltd., Prague, Czech Republic). Homarine content was determined from the peak area, using the equation of linear regression obtained from the calibration curve (concentration vs. optical absorbance at 280 nm) built with five concentrations, in triplicate, with homarine standard.

### 4.4. Cell Culture and Cytotoxicity

RAW 264.7 and AGS cells were maintained in DMEM, while A549 cells were grown in DMEM/F-12, supplemented with 10% heat-inactivated FBS and 1% Pen Strep, and incubated at 37 °C, in a humidified atmosphere of 5% CO_2_ (Toreuse model 2428; St. Louis, MO, USA). RAW 264.7, AGS and A549 cells were seeded in 96-well plates at a density of 35,000, 15,000 and 10,000 cells/well, respectively, and allowed to attach for 24 h. Mitochondrial activity was assessed using the MTT reduction assay, as in Pereira et al. [44]. The extent of the reaction was determined at 560 nm. The results correspond to the mean ± SEM of three independent experiments, each performed in triplicate.

### 4.5. Determination of NO Levels

The ability to interfere with NO levels was assessed according to the procedure described in Ferreres et al. [45]. LPS-induced NO production in RAW 264.7 cells was determined by measuring the level of nitrite accumulation in the culture media by Griess reagent (1% sulfanilamide and 0.1% *N*-[naphth-1-yl]ethylenediamine dihydrochloride in 2% H_3_PO_4_). RAW 264.7 cells were cultured in 96-well plates (35,000 cells/well) for 24 h and then pre-treated with different concentrations of extracts for 2 h. Absorbance was read at 540 nm. Three independent experiments were performed in triplicate, and results are expressed as percentage of NO in cells exposed to LPS (positive control for NO production).

### 4.6. Antimicrobial Activity

Antimicrobial activity of the extracts under study was screened against representative species of bacteria and one yeast grown in Mueller–Hinton broth. The bacteria tested were *Escherichia coli* ATCC 25922, *Staphylococcus aureus* ATCC 25923, *Pseudomonas aeruginosa* ATCC 27853, while the yeast was *Candida albicans* ATCC 10231. Antibacterial activity was performed as we have described before [46], using the latest update to the reference protocol M7-A7 published by the Clinical Laboratory Standards Institute (CLSI, formerly NCCLS). Minimum inhibitory concentration (MIC) was defined as the lowest sample concentration that exhibited complete inhibition of bacterial growth. All determinations were performed in duplicate, and the results were confirmed in three independent assays.

### 4.7. Statistical Analysis

Statistical analysis was performed using GraphPad Prism 6.01 Software (San Diego, CA, USA). One-way analysis of variance (ANOVA with a Tukey’s HSD post hoc test) was used to compare the existence of significant differences amongst the chemical profiles. Concerning the cellular assays, the level of significance between different treatment groups relative to control was determined by one-way analysis of variance (ANOVA), followed by Bonferroni test. Values of *p* ≤ 0.05 were considered statistically significant.

Principal component analysis (PCA) was carried out using IBM SPSS Statistic for Windows version 25.0 (Armonk, NY, USA) and was applied to data obtained from three replicates, to inspect if the ordination of species could be related to fatty acid composition. PCA is a method that indicates (indirect) gradients by producing a smaller set of variables (principal components) that explain the variability of a larger set of variables [47]. PCA was applied to fatty acid composition (μg mg^−1^ dry extract): C12:0, C13:0, C14:1*n*-5c, C14:0, C15:1*n*-5, C15:0, C16:1*n*-7, C16:0, C17:1*n*-7, C17:0, C18:3*n*-6c, C18:2*n*-6c, C18:1*n*-9c, C18:1*n*-9t, C18:0, C20:4*n*-6, C20:5*n*-3, C20:3*n*-6, C20:2*n*-6, C18:3*n*-3c, C20:0, C21:0, C22:6*n*-3, C18:2*n*-6t, C22:1*n*-9, C22:0 and *n*-6/*n*-3.

## 5. Conclusions

This paper constitutes the first report on the chemical characterization of Heterobranchia sea slugs from the genera *Armina* and *Aglaja*. Relevantly, in addition to their chemoecological value, the fatty acid profiles evidenced the production in relevant amounts of specific classes of PUFA, known for their anticancer and anti-inflammatory properties. It is also worth referring to the identification of the alkaloid homarine on the sea slugs under study. Further clues on the metabolic versatility of *A. maculata* and *A. tigrina* as producers of potential anticancer compounds is demonstrated, due to their strong in vitro cytotoxicity towards AGS and A549 cancer cells. Partly associated with the fatty acid profile, namely the high amounts of *n*-3 PUFA, such as eicosapentaenoic (C20:5*n*-3) and docosahexaenoic (C22:6*n*-3) acids, *A. tricolorata* markedly interfered with the NO levels without noticeable toxicity towards RAW 264.7 macrophages, thus supporting its value as a producer of anti-inflammatory metabolites. In light of the results presented in this work, the three Atlantic Heterobranchia sea slugs constitute valuable sources for the discovery of anticancer and anti-inflammatory prototypes.

## Figures and Tables

**Table 1:** Fatty acid content of *A. maculata*, *A. tigrina* and *A. tricolorata* extracts (µg mg^−1^ dry extract) ^1^.

Compound	*A. maculata*		*A. tigrina*		*A. tricolorata*	
	µg mg^−1^ dry extract	Relative %	µg mg^−1^ dry extract	Relative %	µg mg^−1^ dry extract	Relative %
**SFA**						
**C12:0**	nq	nq	nq	nq	-	-
**C13:0**	nq	nq	nq	nq	-	-
**C14:0**	1.20 (0.05)	0.75	1.64 (0.04)	1.20	1.18 (0.16)	0.71
**C15:0**	1.14 (0.04)	0.87	1.14 (0.06)	0.84	1.69 (0.07)	1.02
**C16:0**	30.43 (2.82)	19.08	25.73 (1.10)	18.89	21.28 (1.67)	12.84
**C17:0**	14.60 (0.38)	9.16	13.38 (0.50)	9.82	9.71 (0.61)	5.86
**C18:0**	18.42 (0.49)	11.55	15.20 (0.69)	11.16	15.99 (1.05)	9.64
**C20:0**	1.36 (0.02)	0.85	3.70 (0.11)	2.72	nq	nq
**C21:0**	-	-	nq	nq	-	-
**C22:0**	-	-	2.79 (0.14)	2.05	-	-
**MUFA**						
**C14:1*n*-5**	nq	nq	nq	nq	nq	nq
**C15:1*n*-5**	1.39 (0.14)	0.87	1.48 (0.17)	1.09	-	-
**C16:1*n*-7**	1.72 (0.07)	1.08	2.78 (0.33)	2.04	3.46 (0.17)	2.09
**C17:1*n*-7**	1.13 (0.17)	0.71	1.79 (0.11)	1.32	-	-
**C18:1*n*-9c**	3.91 (0.20)	2.45	3.91 (0.42)	2.87	9.81 (0.64)	5.92
**C18:1*n*-9t**	3.56 (0.28)	2.23	1.71 (0.22)	1.25	7.43 (0.46)	4.48
**C22:1*n*-9**	1.07 (0.14)	0.67	nq	nq	-	-
**PUFA**						
**C18:2*n*-6c**	4.27 (0.27)	2.68	2.36 (0.15)	1.74	3.16 (0.14)	1.91
**C18:2*n*-6t**	-	-	nq	nq	-	-
**C20:2*n*-6**	11.05 (1.23)	6.93	6.68 (0.89)	4.91	19.38 (0.81)	11.69
**C18:3*n*-6c**	nq	nq	nq	nq	2.18 (0.09)	1.31
**C18:3*n*-3c**	4.37 (0.44)	2.74	1.99 (0.24)	1.46	4.20 (0.25)	2.53
**C20:3*n*-6**	5.95 (0.72)	3.73	4.30 (0.78)	3.16	9.63 (0.33)	5.81
**C20:4*n*-6**	30.34 (2.18)	19.03	25.54 (0.93)	18.75	11.38 (0.72)	6.87
**C20:5*n*-3**	20.00 (1.22)	12.54	18.42 (0.87)	13.52	24.02 (1.59)	14.49
**C22:6*n*-3**	3.56 (0.09)	2.23	1.67 (0.13)	1.22	21.28 (1.53)	12.84
**Total**	**159.47 (8.58)**	**100**	**136.21 (6.90)** ^c^	**100**	**165.81 (8.61)**	**100**
**Σ SFA**	67.15 (2.75) ^bbb^	42.11	63.57 (1.44) ^cc^	46.67	49.86 (2.58)	30.07
**Σ MUFA**	12.78 (1.08) ^bbb^	8.01	11.67 (1.04) ^ccc^	8.57	20.71 (1.20)	12.49
**Σ PUFA**	79.55 (6.11) ^a,b^	49.88	60.97 (4.41) ^cc^	44.76	95.24 (4.92)	57.44
**Σ** ***n*-3**	27.93 (1.83) ^bbb^	17.51	22.07 (0.98) ^cccc^	16.21	49.51 (2.85)	29.86
**Σ** ***n*-5**	1.39 (0.14) ^bb^	0.87	1.48 (0.17) ^cc^	1.09	nq	-
**Σ** ***n*-6**	51.62 (4.28) ^a^	32.37	38.89 (3.44)	28.55	45.74 (2.07)	27.59
**Σ** ***n*-7**	2.86 (0.20) ^aaa,b^	1.79	4.57 (0.23) ^cc^	3.36	3.46 (0.17)	2.09
**Σ** ***n*-9**	8.53 (0.59) ^a,bbb^	5.35	5.62 (0.65) ^cccc^	4.12	17.24 (1.07)	10.40
***n*-6/*n*-3**	1.85 (0.03) ^bbbb^		1.76 (0.08) ^cccc^		0.92 (0.01)	

^1^ Results are expressed as the mean (standard deviation) of a triplicate analysis. nq: not quantified; “-“ not detected; *∑* sum. Comparison between: *A. maculata* and *A. tigrina*, ^a^ *p* < 0.05 and ^aaa^ *p* < 0.001. Comparison between *A. maculata* and *A. tricolorata*: ^b^ *p* < 0.05, ^bb^ *p* < 0.01, ^bbb^ *p* < 0.001 and ^bbbb^ *p* < 0.0001. Comparison between *A. tigrina* and *A. tricolorata*: ^c^ *p* < 0.05, ^cc^ *p* < 0.01, ^ccc^ *p* < 0.001, and ^cccc^ *p* < 0.0001.

**Table 2:** Homarine content of *A. maculata*, *A. tigrina* and *A. tricolorata* extracts (µg g^−1^ dry extract) ^1^.

Compound	*A. maculata*	*A. tigrina*	*A. tricolorata*
Homarine	969.74 (43.90) ^aaaa,bbb^	nq ^cccc^	1237.15 (35.08)

^1^ Results are expressed as the mean (standard deviation) of three determinations. nq: not quantified. Comparison between *A. maculata* and *A. tigrina*: ^aaaa^ *p* < 0.0001. Comparison between *A. maculata* and *A. tricolorata*: ^bbb^ *p* < 0.001. Comparison between *A. tigrina* and *A. tricolorata*: ^cccc^ *p* < 0.0001.

## Abbreviations

The following abbreviations are used in this manuscript:

ATCC	American Type Culture Collection
CLSI	Clinical Laboratory Standards Institute
DAD	Diode-array detector
DMSO	Dimethyl sulfoxide
DMEM	Dulbecco’s modified Eagle medium
ECACC	European Collection of Authenticated Cell Cultures
FAME	Fatty acid methyl esters
FBS	Fetal bovine serum
GC-FID	Gas-chromatography-Flame Ionization Detection
HPLC	High-performance liquid chromatography
IC_50_	Half maximal inhibitory concentration
LPS	Lipopolysaccharide
MIC	Minimum inhibitory concentration
MTT	Thiazolyl blue tetrazolium bromide
MUFA	Monounsaturated fatty acids
NO	Nitric oxide
PCA	Principal Component Analysis
PPAR	Peroxisome proliferator-activated receptors
PUFA	Polyunsaturated fatty acids
ROS	Reactive oxygen species
SEM	Standard error of the mean
SFA	Saturated fatty acids
UFA	Unsaturated fatty acids
